# Metabolic footprint of aging and obesity in red blood cells

**DOI:** 10.18632/aging.202693

**Published:** 2021-02-19

**Authors:** Inés Domingo-Ortí, Rubén Lamas-Domingo, Andreea Ciudin, Cristina Hernández, José Raúl Herance, Martina Palomino-Schätzlein, Antonio Pineda-Lucena

**Affiliations:** 1 Drug Discovery Unit, Instituto de Investigación Sanitaria La Fe, Valencia 46026, Spain; 2 NMR Facility, Centro de Investigación Príncipe Felipe, Valencia 46012, Spain; 3 Diabetes and Metabolism Research Unit, Vall d’Hebron Research Institute, Barcelona 08035, Spain; 4 CIBERDEM (Instituto de Salud Carlos III), Madrid 28029, Spain; 5 Medical Molecular Imaging Research Group, Vall d’Hebron Research Institute, CIBBIM-Nanomedicine, Barcelona 08035, Spain; 6 CIBERBBN (Instituto de Salud Carlos III), Madrid 28029, Spain; 7 Medicinal Chemistry Laboratory, Centro de Investigación Médica Aplicada, Pamplona 31008, Spain

**Keywords:** RBCs, NMR, metabolomics, aging, obesity

## Abstract

Aging is a physiological process whose underlying mechanisms are still largely unknown. The study of the biochemical transformations associated with aging is crucial for understanding this process and could translate into an improvement of the quality of life of the aging population. Red blood cells (RBCs) are the most abundant cells in humans and are involved in essential functions that could undergo different alterations with age. The present study analyzed the metabolic alterations experienced by RBCs during aging, as well as the influence of obesity and gender in this process. To this end, the metabolic profile of 83 samples from healthy and obese patients was obtained by Nuclear Magnetic Resonance spectroscopy. Multivariate statistical analysis revealed differences between Age-1 (≤45) and Age-2 (>45) subgroups, as well as between BMI-1 (<30) and BMI-2 (≥30) subgroups, while no differences were associated with gender. A general decrease in the levels of amino acids was detected with age, in addition to metabolic alterations of glycolysis, the pentose phosphate pathway, nucleotide metabolism, glutathione metabolism and the Luebering-Rapoport shunt. Obesity also had an impact on the metabolomics profile of RBCs; sometimes mimicking the alterations induced by aging, while, in other cases, its influence was the opposite, suggesting these changes could counteract the adaptation of the organism to senescence.

## INTRODUCTION

Changes in life expectancy due to better living conditions and healthcare, together with a decrease in birth rates, are resulting in an increasing aging population [1]. The aging process induces significant alterations in the human body and is characterized by many transformations, ranging from organs to cellular organelles, the whole process leading to a wide variety of modifications of biological functions (sensory, physiological, cognitive, physical, etc.). Aging is influenced by genetic and environmental factors [2] and has been associated with many pathological processes [3, 4]. In this context, to ensure a healthy environment and a good quality of life for aging population, it is of paramount importance to get a better understanding of the precise physiology of aging, a process that is still not well understood.

Many of the changes associated with aging, including free radical generation, advanced glycoxidation, end products formation, lipid peroxidation or inflammatory responses, are linked to metabolism [5]. Therefore, the evaluation of global changes in metabolism could provide a valuable tool to advance in the understanding of this biological process. The metabolome, defined as the collection of small molecules characterizing a biological system, is the downstream result of genomic and proteomic activity, and provides important insights into the physiological regulation of the human body [6]. The personalized analysis of the metabolome for each individual informs about his/her genetics, lifestyle, age and environmental factors [7], and it is a promising tool for elucidating the mechanisms responsible for diverse physiological and pathophysiological states by exploring and integrating information from multiple pathways and networks [8].

Age-related metabolic alterations in humans have already been characterized in different biofluids, mostly in blood [9–22] and urine [16, 20, 23]). Metabolomic studies have been usually performed in plasma or serum samples [24–32] as they contain a high number of metabolites, and can be easily obtained and preserved. However, the metabolomic signature of the cellular blood fraction, that contains relevant information on the physiological condition of the organism [33], has so far been poorly assessed [12, 18].

Red Blood Cells (RBCs) are the most abundant cells in blood and contain a large amount of metabolites, providing a relevant source of information on the physiological status of an individual. The main biological function of RBCs is the transport of oxygen to body cells and the delivery of carbon dioxide to the lungs [34]. As they constitute an integral component of the blood, RBCs facilitate the transport of metabolites between organs, and its analysis could contribute to get a better understanding of the metabolic situation of the whole organism. RBCs possess their own metabolism, a relatively simple one due to the absence of nuclei and organelles [33]. Therefore, they exhibit a different metabolomic signature from the acellular blood fraction, making its study even more relevant. Furthermore, RBCs are abundant, easily accessible through standard blood analysis, and very sensitive to oxidative stress [35]. However, and despite its informative potential, metabolomics research on RBCs has been very limited.

Obesity is experiencing a worldwide epidemic increase and has become a major social and public health problem. According to the World Health Organization (WHO), in 2016, more than 1900 million adults were found to be overweight, and 650 million of them were obese [36]. Besides the well-known impact on metabolic and cardiovascular morbidity and mortality, obesity is associated with major physical, psychosocial, psychological and occupational complications that contribute to worsening individuals' quality of life and decreasing their life expectancy [37]. High body mass indexes (BMIs) have also been associated with diseases whose incidence increases with age (e.g., type II diabetes mellitus, cardiovascular disease, liver and kidney dysfunctions, hypertension, depression, musculoskeletal and respiratory disorders and some neoplastic processes) [38, 39]. In a study focused on the interactions between the aging hallmarks and obesity, Salvestrini et al. found that an excess of nutrients provokes cellular responses that lead to obesity, and ultimately contributes to an increase of the aging rate [40]. In fact, several alterations caused by obesity have also been found during aging, such as the accumulation of fat mass due to a decline on energy expenditure [41] and endocrinological changes, including decreased hormone levels and insulin and leptin resistance [42]. Therefore, it would be relevant to assess the metabolic signatures of both obesity and aging for getting a better understanding of potential common features.

A significant number of studies, based on the metabolomics analysis of plasma/serum [19, 43–50] and urine [48, 50, 51], have already been carried out on the metabolomics alterations associated with obesity. However, only few of them have used RBCs for the evaluation of these metabolic disorders. Bird et al. found increased folate levels in RBCs from obese patients [52] and Del Genio et al. carried out an analysis of the fatty acid composition of the membrane of RBCs of these individuals [53].

The impact of aging and obesity is different in women and men [54]. Gender has a strong impact on metabolism [55–58] and several studies have focused on the analysis of the differences in the metabolite composition of blood from women and men (plasma/serum [19, 21, 22, 49, 59–61]) and RBCs [62, 63]. However, although some of these studies have evaluated the effect of aging [22] and obesity [46], none of them have analyzed the metabolite content of RBCs.

In this context, the aim of this work was to evaluate the impact of aging on the metabolomics profile of RBCs obtained from a cohort of healthy individuals using Nuclear Magnetic Resonance (NMR) spectroscopy. The alterations of these age-specific metabolomics profiles in obese and morbid-obese individuals were also studied. Finally, gender-specific differences in the age- and weight-associated metabolic changes of these individuals were characterized. Overall, we expect this study could contribute to get a better understanding of how RBCs could be used to analyze the metabolic changes associated with different physiological situations.

## RESULTS

### Metabolomics profile of RBCs

**Figure 1:**
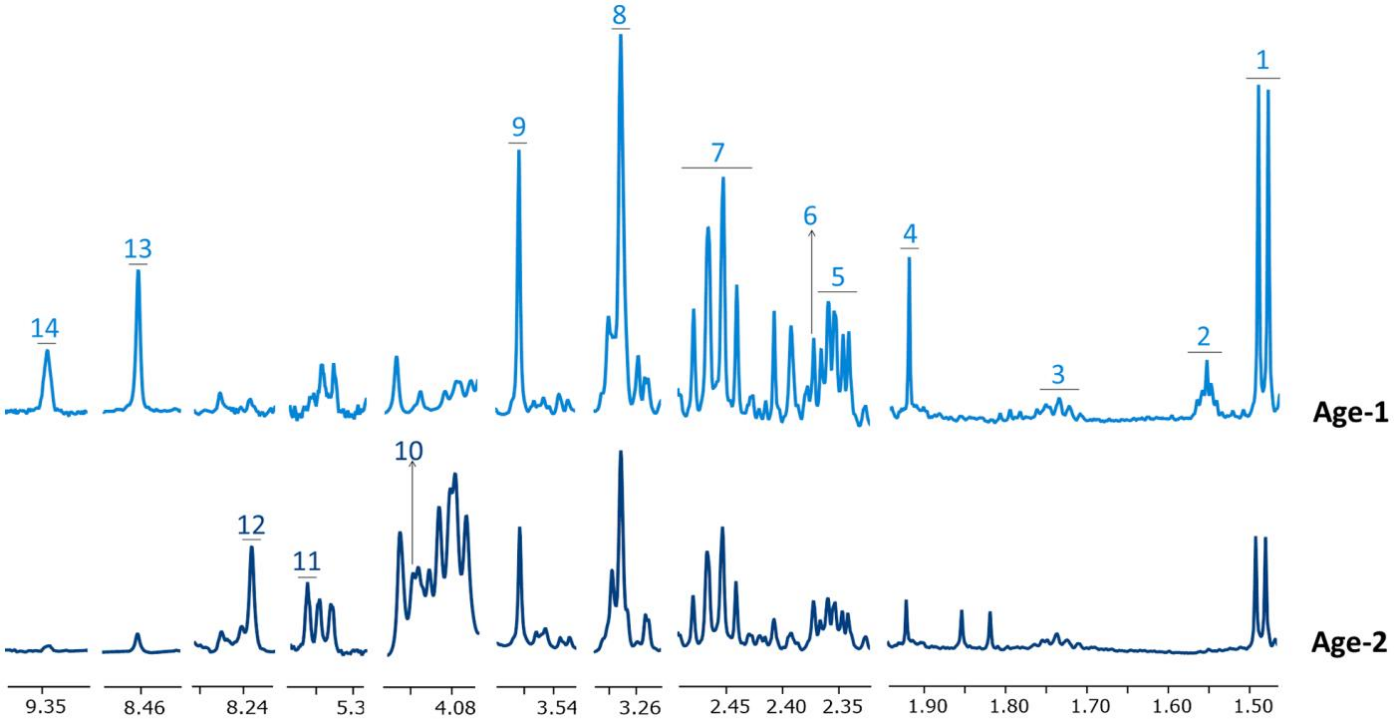
**Representative ^1^H-NMR spectra corresponding to Age-1 and Age-2 groups.** Spectral regions are widened to better appreciate changes in the levels of some metabolites. Metabolites in Age-1 and Age-2 spectra are negatively and positively associated with age, respectively. Assigned metabolites: *1* alanine, *2* adipate, *3* lysine, *4* acetate, *5* glutamate, *6* pyruvate, *7* glutamine, *8* betaine, *9* glycine, *10* 6-phosphogluconate, *11* phosphoenolpyruvate, *12* inosine monophosphate, *13* formate, *14* NAD+.

Good quality 1H-NMR spectra were obtained for the RBC extracts from the peripheral blood of subjects included in Table 1. Figure 1 and Supplementary Figure 1 display representative spectra from the Age-1 (≤45 years) and the Age-2 (>45years) groups. The spectra had a good signal-to-noise ratio and allowed the detection and quantification of 55 different metabolites, as summarized in Supplementary Table 1. Chemical shift assignment of the metabolites was confirmed by metabolite spiking experiments (Supplementary Figure 2 and Supplementary Table 2) and 2D NMR experiments (Supplementary Figure 3 and Supplementary Table 2). It should be noted that a high number of those metabolites, including 2,3-biphosphoglycerate (2,3-BPG), 3-methyladipate, 6-phosphogluconate (6-PG), adenosine, adipate, AMP, ATP, choline, fumarate, glucose-1-phosphate (G1P), glutathione, inosine monophosphate (IMP), Nicotinamide adenine dinucleotide (NAD+), Nicotinamide adenine dinucleotide phosphate (NADP+), niacinamide, phosphoenolpyruvate, phosphocreatine, propylenglycol, succinate and UDP-glucose are not usually detected in human serum/plasma by NMR [64, 65].

**Table 1:** Characteristics of the individuals included in the study.

**Classification**	**Range**	**Subject number**	Age*	BMI*	**M/W**
All	-	83	48.12 ± 1.56	30.85 ± 1.05	37/46
Age-1	≤45	31	34.07 ± 1.57	29.10 ± 1.40	11/20
Age-2	>45	31	51 ± 1.70	29.80 ± 0.97	10/21
A1	19-40	26	27.84 ± 1.30	22.30 ± 0.66	12/14
A2	40-60	13	51.00 ± 1.70	25.32 ± 0.93	8/5
A3	60-75	11	65.09 ± 1.12	26.91 ± 1.19	7/4
BMI-1	<30	39	50.93 ± 2.17	24.00 ± 0.58	22/17
BMI-2	≥30	35	51.67 ± 1.70	39.10± 1.17	12/23
B1	<30	17	51.35 ± 3.09	24.77 ± 0.83	9/8
B2	30-40	17	51.71 ± 2.92	33.60 ± 0.73	8/9
B3	>40	16	51.35 ± 2.04	45.43 ± 0.79	3/13
M	-	28	49.79 ± 2.65	33.46 ± 1.80	-
W	-	28	49.43 ± 2.72	30.65 ± 1.30	-

(*): Age and BMI values are expressed as mean ±SEM (Standard Deviation of the Mean). M: Men. W: Women.

A principal component analysis (PCA) was performed for all samples included in the study (Supplementary Figure 4) to identify potential outliers. This analysis revealed the existence of three samples located outside the 95% Hoteling’s T-square. However, the spectra of these samples did not exhibit any abnormal features. Therefore, they were not excluded from the study as they could represent biological variation within the group of samples.

The PCA score plot was colored according to the age (Age-1 (≤45) vs Age-2 (>45), weight (BMI-1 (<30) vs BMI-2 (≥30)) and gender (M vs W) of the individuals to detect potential trends in the data according to these variables (Supplementary Figure 4). However, no clear separation between the subgroups could be observed. Therefore, discriminant analyses were pursued to evaluate the specific differences due to the age, BMI or gender subgroups of the individuals.

### Age has a relevant impact on the metabolomic profile of RBCs

**Figure 2:**
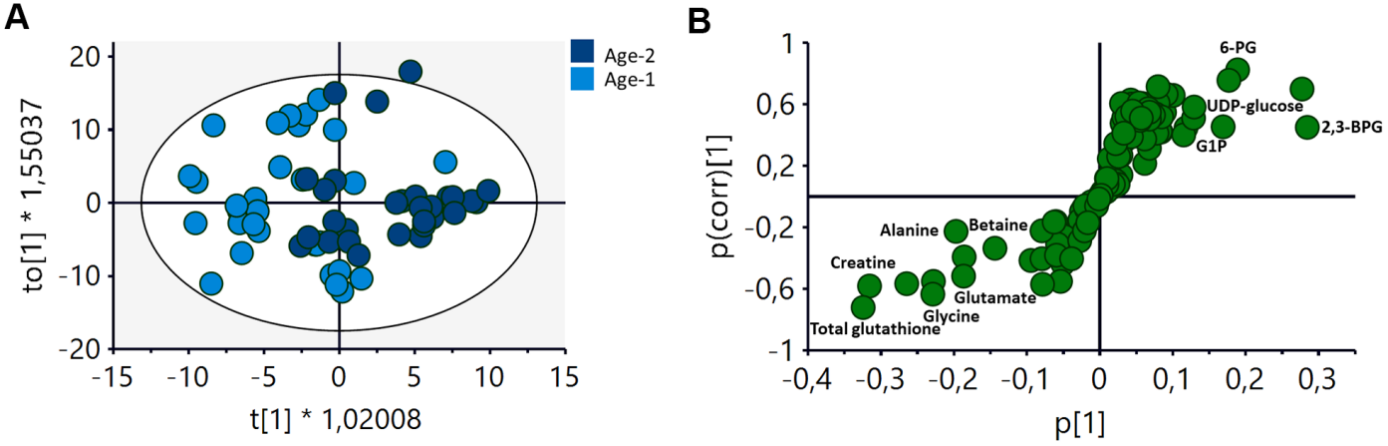
**OPLS-DA analysis of the metabolomic profile of RBCs of Age-1 (≤45 years) and Age-2 (>45 years) groups.** (**A**) Score plot of the OPLS-DA model obtained. R2Y(cum)=0,675, Q2(cum)=0.208. Permutation test result: R2=(0.0, 0.249), Q2=(0.0, -0.276). CV-Anova: p-value=0.0388. (**B**) S-plot showing the most important metabolites contributing to the discrimination between the Age-1 and Age-2 groups. 2,3-BPG: 2,3-biphosphglycerate, 6-PG: 6-phosphogluconate, G1P: glucose 1-phosphate.

An initial evaluation of the impact of age on the metabolic profile of RBCs was derived from the analysis of an OPLS-DA (orthogonal projection to latent structures discriminant analyses) comparing samples from the BMI-matched Age-1 and Age-2 groups. Using this approach, a discriminant model was obtained (R2Y(cum)=0.675, Q2(cum)=0.208), showing a different metabolic signature for the two groups (Figure 2A). The corresponding S-plot (Figure 2B) showed that an increase in metabolites associated with the metabolism of carbohydrates and a decrease in the levels of certain amino acids and glutathione were partially responsible for the discrimination between both age groups.

**Figure 3:**
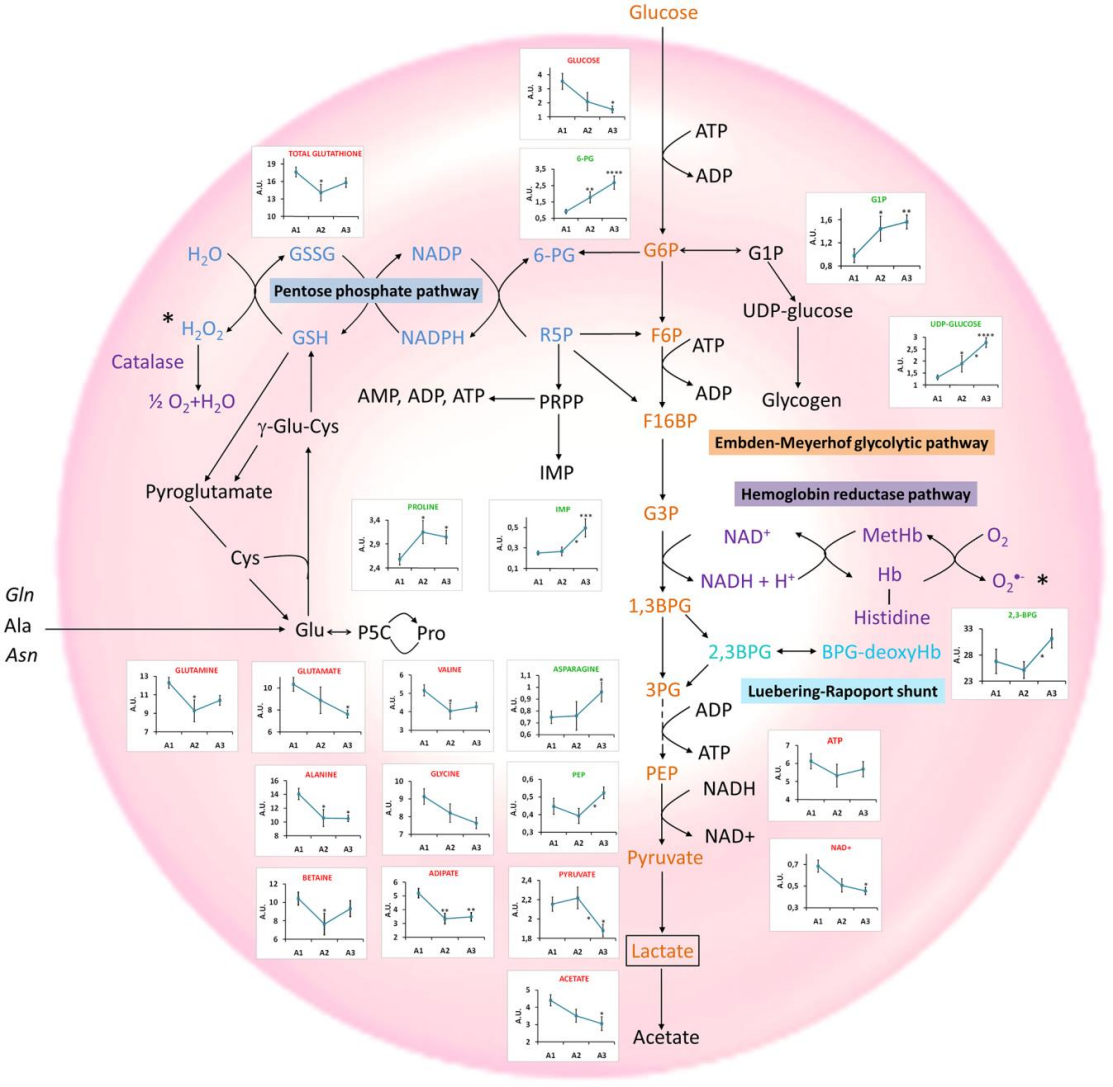
**Main metabolomic pathways found in RBCs and summary of the most relevant age-associated metabolic alterations.** Concentration values are normalized to total intensity. A1=between 19 and 40 years, A2 = between 40 and 60 years; and A3≥60years. Values are represented as mean±SEM. * p < 0.05, ** p < 0.01, *** p < 0.001, **** p < 0.0001. P-values were calculated with a Student’s t-test. 1,3BPG: 1,3-biphosphoglycerate, 2,3BPG: 2,3-biphosphoglycerate, 3PG: 3-phosphoglycerate, 6-PG: 6-phosphogluconate, ADP: adenosine diphosphate, Ala: alanine, Asn: Asparagine, ATP: adenosine triphosphate, BPG-deoxyHb: biphosphoglycerate-deoxyhemoglobin, Cys: cysteine, F6P: fructose 6-phosphate, F16BP: fructose 1,6-biphosphate, G6P: glucose 6-phosphate, G1P: glucose 1-phoshate, G3P: Glyceraldehyde 3-phosphate, Glu: glutamate, Gln: glutamine, GSH: reduced glutathione, GSSG: oxidized glutathione, IMP: inosine monophosphate, MetHb: methaemoglobin, NAD+: Nicotinamide adenine dinucleotide, NADP+: Nicotinamide adenine dinucleotide phosphate, P5C: 1-pyrroline-5-carboxylate, PEP: phosphoenopyruvate, Pro: proline, PRPP: phosphoribosyl pirophosphate, R-5-P: ribose 5-phosphate.

To further characterize the specific changes associated with age, an univariate statistical analysis of metabolites exhibiting VIP (variable importance for projection) values > 1 was also carried out. Figure 3 and Supplementary Table 3 summarize the results obtained based on the changes observed for different age-range groups (19<A1<40 years, 40<A2<60 years and 60<A3<75), as well as a graphical representation of the main metabolic pathways of RBCs. This analysis revealed that several metabolites from the Embden-Meyerhof glycolytic pathway, the pentose phosphate pathway (PPP), the nucleotide metabolism, the glutathione metabolism as well as the Luebering-Rapoport shunt experienced important alterations with age. Furthermore, a general decrease in the levels of many amino acids was also detected, except for proline and asparagine that showed the opposite trend. Finally, the levels of adipate and betaine were found to be negatively correlated with age.

This evaluation also revealed that the metabolic alterations involving amino acids and glutathione were associated with changes at early ages, as the most important differences in the levels of these metabolites were found between the A1 and A2 groups. Interestingly, the changes associated with the glycolysis, the PPP or the Luebering-Rapport shunt were found to be more significant only when comparing the A2 and A3 groups.

### Obesity plays a role in the metabolic composition of RBCs

**Figure 4:**
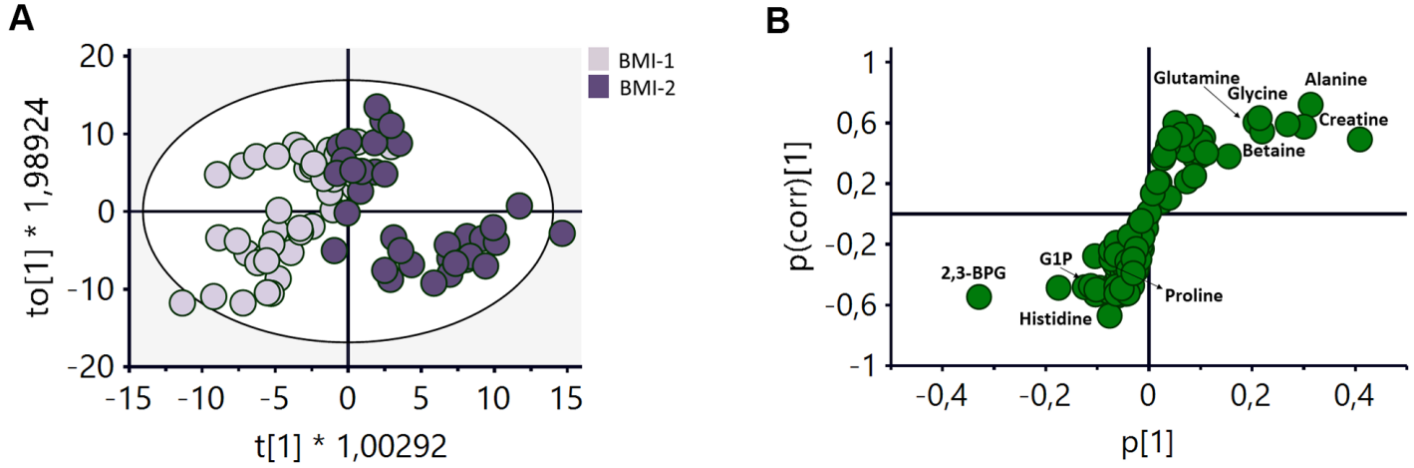
**OPLS-DA analysis of the metabolomic profile of RBCs of BMI-1 (BMI<30) and BMI-2 (BMI≥30) groups.** (**A**) Score plot of the OPLS-DA model obtained. R2Y(cum)=0.75, Q2(cum)=0.413. Permutation test result: R2=(0.0, 0.248), Q2=(0.0, -0.362). CV-Anova: p-value=0.000016. (**B**) S-plot showing the most important metabolites contributing to the discrimination between non-obese and obese subjects. G1P: glucose 1-phoshate, 2,3BPG: 2,3-biphosphoglycerate.

The effect of BMI on the metabolomic profile of RBCs was also examined. Individuals were classified into non-obese (BMI-1< 30) and obese (BMI-2 ≥ 30) to carry out this analysis. An OPLS-DA analysis of these two groups (Figure 4A, R2Y(cum)=0.75, Q2(cum)=0.413) revealed the existence of a number of metabolites contributing to the discrimination between these two groups (Figure 4B). An increase of different amino acids (glutamine, glycine and alanine), creatine, and betaine, as well as a decrease of histidine, proline, G1P and 2,3-BPG appeared to be the main changes responsible for the discrimination between both groups.

**Figure 5:**
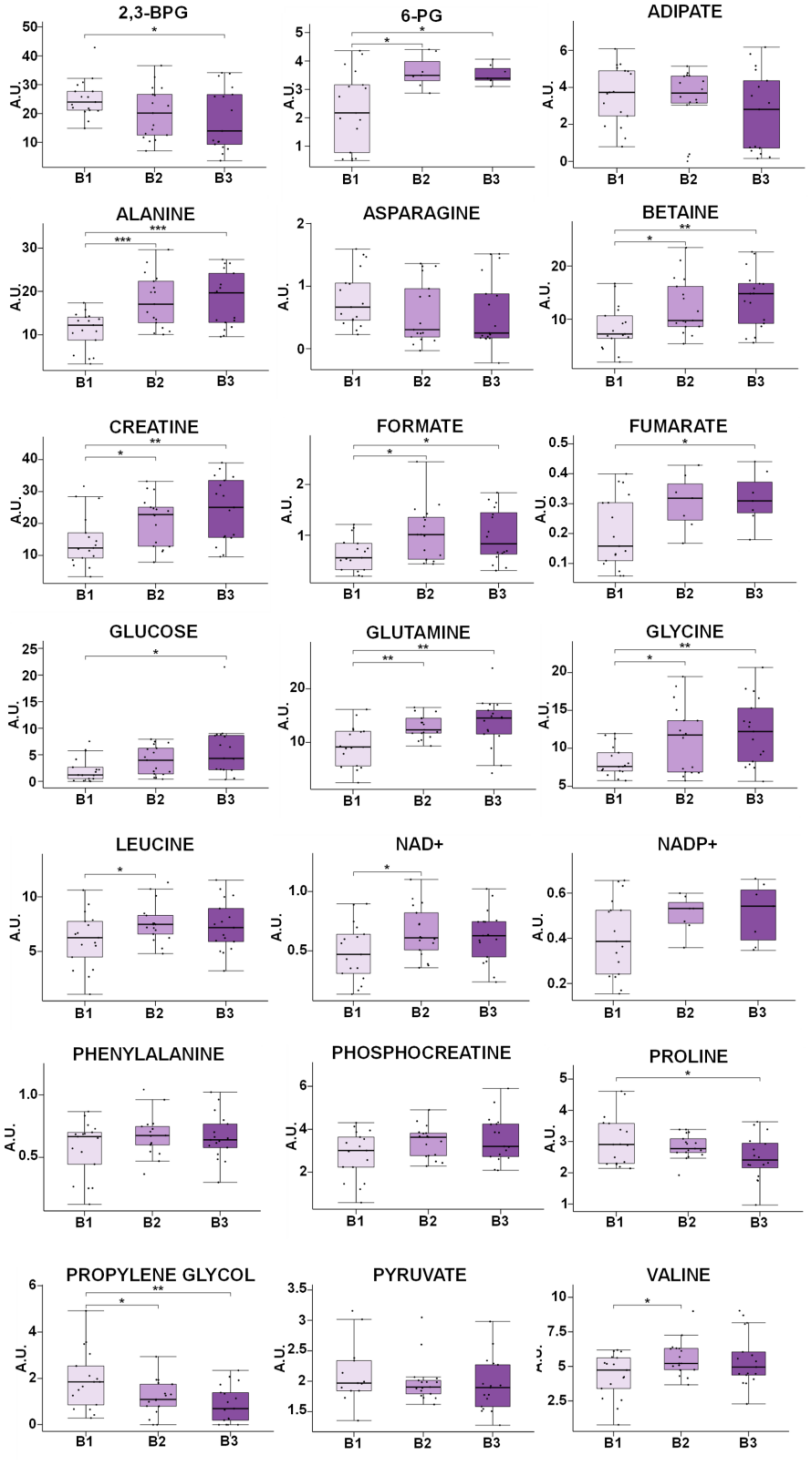
**Box-plot comparison of the concentrations associated with the most relevant metabolites involved in the discrimination based on the BMI value.** Metabolites that do not present statistically significant changes, but show clear trends with BMI, has been also included (NADP+, phenylalanine, phosphocreatine, asparagine). Concentration values are normalized to total intensity. Values are represented as mean±SEM. * p < 0.05, ** p < 0.01, *** p < 0.001. P-values were calculated with a Student’s t-test. For each box, the central line is the median, the edges of the box are the upper and lower quartiles, the whiskers extend the box by a further ±1.5 interquartile range (IQR) and outliers are plotted as individual points. 6-PG: 6-phosphogluconate, NAD+: Nicotinamide adenine dinucleotide NADP+: Nicotinamide adenine dinucleotide phosphate, 2,3-BPG: 2,3-biphosphoglycerate, G1P: Glucose 1-phosphate.

To further evaluate the alterations associated with obesity, univariate analyses were also carried out for all the metabolites assigned. The levels of most of them (6-PG, alanine, betaine, creatine, fumarate, glucose, glutamine, glycine, leucine, formate, valine and NAD+) were positively correlated with BMI, and some of them (2,3-BPG, G1P, histidine, proline and propylene glycol) showed the opposite trend. Figure 5 includes the box-plots of metabolites exhibiting clear, albeit not significant, trends with BMI. This analysis was based on a classification of the samples on three subgroups according to the BMI values (B1, B2 and B3) of the individuals. Most amino acids showed a positive correlation with BMI, except for proline and asparagine that exhibited an opposite trend, in contrast with the behavior observed for their variations with age. Glucose levels were found to be increased in obese individuals, as well as the levels of NAD+ and NADP+, formate, fumarate and betaine. On the other hand, 2,3-BPG and propylene glycol levels were negatively correlated with BMI.

In general, the most significant changes (Figure 5 and Supplementary Table 4) were found for the comparison between non-obese (B1) and obese subjects (B2), and only some marginal alterations were observed when comparing obese (B2) and morbid obese individuals (B3).

### Gender does not have a significant impact on the metabolomics profile of RBCs

**Figure 6:**
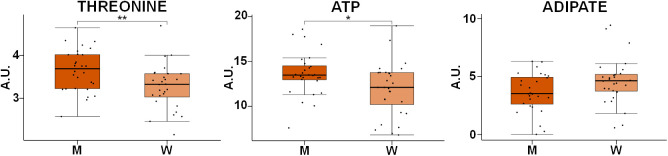
**Box-plot comparison of the concentrations associated with the most relevant metabolites involved in the discrimination based on gender.** Concentration values are normalized to total intensity. Values are represented as mean±SEM. * p < 0.05, ** p < 0.01, *** p < 0.001, **** p < 0.0001. P-values were calculated with a Student’s t-test. For each box, the central line is the median, the edges of the box are the upper and lower quartiles, the whiskers extend the box by a further ±1.5 interquartile range (IQR) and outliers are plotted as individual points. M: men, W: women, ATP: adenosine triphosphate.

The effect of the gender of the individuals on the metabolic profile of RBCs was also assessed. To this end, an OPLS-DA analysis of age- and BMI-matched gender groups was performed. Interestingly, no significant model could be generated for the comparison between both groups, suggesting that the impact of gender on the metabolomics profile of RBCs is less pronounced than that of age or weight. Despite the negative result obtained for the multivariate analysis, an additional univariate analysis was carried out to evaluate each metabolite individually. None of the metabolites experiencing significant changes with age and BMI were influenced by gender. However, the univariate analysis of all the metabolites identified in RBCs showed significant statistical differences in the levels of threonine and ATP and a clear trend in adipic acid concentrations between men (M) and women (W) (Figure 6).

### Correlation between the metabolomic profile of RBCs with age and weight

To further evaluate the effect of age and weight on the metabolomic profile of RBCs, different partial least squares (PLS) regression analyses were carried out based on these two variables. The PLS analysis versus age was carried out separately for the BMI-1 and BMI-2 groups, while the PLS analysis versus BMI was carried out for the Age-1 and Age-2 groups, to avoid interferences between both parameters as they both had an effect on the metabolomic signature of RBCs.

**Figure 7:**
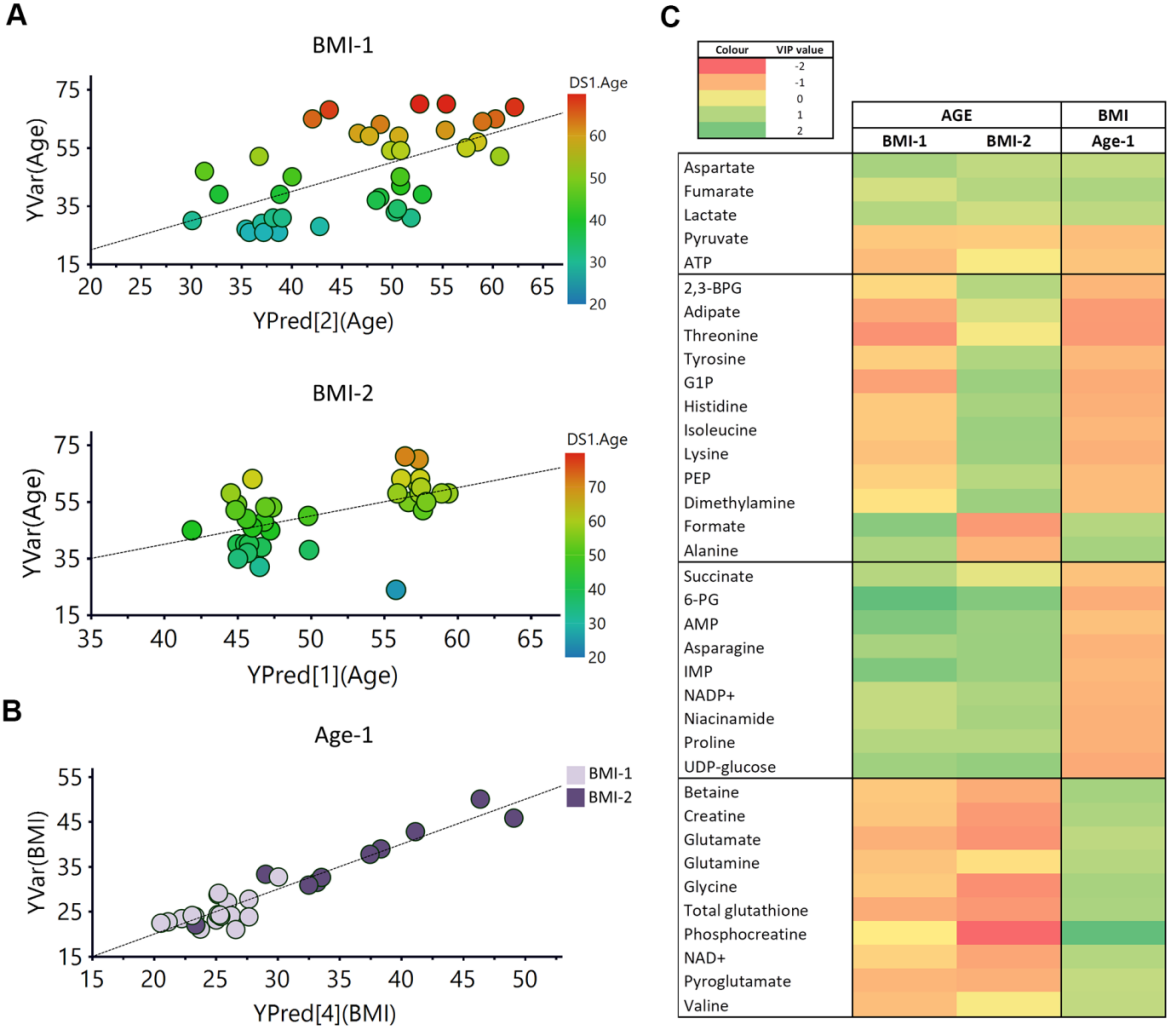
**PLS analysis vs age and BMI.** (**A**) PLS model vs age in BMI-1 group (BMI<30; R2Y(cum)= 0.355, Q2(cum)=0.207), Permutation test result: R2=(0.0, 0.261), Q2=(0.0, -0.162), p from CV-ANOVA =0.084) and in BMI-2 subjects (BMI≥30; R2Y(cum)= 0.294, Q2(cum)=0.185), Permutation test result: R2=(0.0, 0.149), Q2=(0.0,-0.0799), p from CV-ANOVA =0.038). (**B**) PLS model vs BMI for Age-1 individuals. R2Y(cum)= 0.9, Q2(cum)= 0.649, p= 0.00036, Permutation test result: R2=(0.0, 0.647), Q2=(0.0,-0.286), p from CV-ANOVA =0.0041 (**C**) Heatmap representation of the metabolites with variable importance in projection (VIP) values > 1 of the PLS regression models vs age or BMI. 2,3-BPG: 2,3-biphoshoglycerate, 6-PG: 6-phosphogluconate, AMP: adenosine monophosphate, ATP: adenosine triphosphate, G1P: glucose 1-phosphate, IMP: inosine monophosphate, NAD+: Nicotinamide adenine dinucleotide, PEP: phosphoenolpyruvate.

Regression models based on the age of the individuals were obtained for both BMI groups, and validated by cross validation and permutation (Figure 7A). However, for the PLS regression analysis as a function of the BMI values, a model could only be obtained for the Age-1 subgroup (individuals ≤45 years) (Figure 7B). Interestingly, the correlation obtained as a function of the BMI values was more robust than that obtained for age. Metabolites exhibiting significant VIP values are summarized in Figure 7C. In this figure, the first section refers to metabolites following the same general trend with age and BMI, and the second block includes metabolites sharing the same trend for the BMI-1 subgroup (age) and the Age-1 subgroup (BMI), which is exactly the opposite behavior of the BMI-2 subgroup (age). The third and fourth blocks display metabolites exhibiting opposite trends with age and BMI. As shown, many metabolites underwent similar changes in both age models (BMI-1 and BMI-2). However, for some metabolites, changes were different for both groups, including several amino acids, 2,3-BPG, G1P, adipate and formate. Interestingly, some other metabolites followed a similar trend for BMI and age (e.g., aspartate, ATP and some organic acids -fumarate, lactate and pyruvate-). Furthermore, the results suggested that age induced a similar effect on non-obese (BMI-1) individuals than BMI on younger individuals (Age-1). In other cases, changes associated with age and weight exhibited opposite trends (e.g. creatine, betaine and several amino acids, nucleotides and carbohydrates).

## DISCUSSION

RBCs play a central role in human physiology by delivering oxygen and nutrients to the body cells. To carry out these activities, RBCs have to preserve their integrity by maintaining active an antioxidant defensive mechanism based on the glutathione redox system. In these circumstances, the analysis of the metabolic profile of RBCs not only provides information on the compounds transported by these cells in blood, it also informs on their own metabolic routes (glycolysis, Luebering-Rapoport shunt, fatty acid synthesis, nucleotide pathways, etc.) [66, 67]. Specifically, the characterization of the metabolomics profile of RBCs offers an opportunity to obtain relevant information, complementary to that provided by the acellular blood fraction [68], on the metabolic impact of different physiological processes. In this context, the focus of this study was to evaluate, for the first time, the impact of age, BMI and gender on the metabolomics profile of RBCs.

A discriminant multivariate analysis carried out to assess the impact of age on the metabolic profile of RBCs revealed that it was possible to obtain a statistically significant discriminant model when comparing the Age-1 (≤45 years) and Age-2 (>45 years) subgroups (Figure 2A), as it was suggested by Chaleckis et al. [18]. The main metabolites contributing to this discrimination were associated with the glycolytic pathway (6-PG, UDP-glucose, G1P and 2,3-BPG), as well as with alterations in the levels of certain amino acids (alanine, betaine, creatine, glycine and glutamate) and glutathione (Figure 2B).

An in-depth evaluation of the results, based on the univariate analysis of different age-range groups (Figure 3), revealed a significant decrease in the levels of pyruvate and NAD+, reflecting a down-regulation of the Embden-Meyerhof glycolytic pathway, as it was previously reported [69]. Reduced levels of acetate were also found as age increased, in agreement with the down-regulation of the glycolytic pathway. However, the lactate pool, end product of this pathway, exhibited a slight increase with age (Supplementary Table 3), perhaps reflecting that lactate cannot be further metabolized in the erythrocytes and is released to the general circulation. A correlation between elevated levels of lactate and age has also been found in brain studies [70], and associated with the onset of some diseases [71].

Age also had a strong impact on the PPP (Figure 3), as it is also shown in the pathway analysis performed with Metaboanalyst [72] (Supplementary Figure 5A). One of the main purposes of this pathway is to generate NADPH, a cofactor involved in the regeneration of reduced glutathione, necessary to prevent damage caused by oxidative stress on RBCs [73]. 6-PG, a key metabolite of this route, showed a statistically significant positive correlation with age [69]. This fact could be associated with a lack of glucose 6-phosphate isomerase, the enzyme that catalyzes the interconversion of glucose 6-phosphate (G6P) and fructose 1-phosphate (F1P), channeling G6P to the PPP pathway [74].

The results did not reveal any decrease of GSH levels with age. However, as GSH is easily oxidized to GSSG, the study of total glutathione in RBCs could perhaps provide a better reflection of the general redox status of the cells with age. This analysis revealed that the levels of total glutathione (GSH+GSSG) decreased with age (Figure 3 and Supplementary Figure 5A), as reported in previous studies based on the analysis of RBCs [12, 75]. This decrease would explain, at least partially, the damage suffered by RBCs with aging, since it is the primary antioxidant mechanism operating in RBCs. Interestingly, the precursors glycine, glutamine and glutamate [76] and the NAD+ pool [77] were also decreased (Figure 3), affecting glutamine and glutamate metabolism (Supplementary Figure 5A). Reduced NAD+ and NADP+ levels in blood plasma have been previously associated with aging [18, 44]. The concentration of these metabolites in RBCs is higher, being easier to detect by NMR than in plasma samples, where these signals overlap with the signals of proteins present in blood. For this reason, NAD+ blood levels are usually measured using other experimental approaches, such as LC-MS [78], enzymatic assays [79] or biosensors [80]. Therefore, the analysis of RBCs could be a complementary tool to standard blood plasma analysis for the determination of these molecules that are somehow associated with biological aging.

The PPP pathway is connected to the nucleotide metabolism through the PRPP (phosphoribosyl pyrophosphate) synthesis, involved in the generation of ATP and IMP. The study revealed an increase of the IMP levels, as well as a decrease, although not significant, of the ATP levels, as age increased, perhaps reflecting an alteration of the PPP pathway that would eventually lead to changes in the nucleotide metabolism (Figure 3). Moreover, the levels of G1P and UDP-glucose, precursors of the synthesis of glycogen, experienced a significant increase with age (Figure 3), suggesting an accumulation of these molecules as a consequence of a decreased glycogen synthesis capacity due to the lower activity of glycogen synthase with age [81].

Furthermore, a group of metabolites transported by RBCs were found to experience significant alterations with age (Supplementary Figure 5A). Among them, almost all the amino acids showed a decrease with age (Figure 3), a process that has already been reported in previous studies based on the analysis of serum and plasma samples [10, 13, 18, 20, 49, 82, 83]. In particular, a decrease in the concentrations of branched-chain amino acids (BCAAs: valine, leucine and isoleucine) confirmed other studies performed in serum and plasma [18, 84, 85]. In addition to BCAAs, other essential (lysine, threonine) and non-essential amino acids (alanine, glycine, glutamine) exhibited a significant decrease with age, as it has been found in other studies performed on RBCs [12] and serum [22, 36, 71, 72]. The reduction of glutamine levels has been observed in RBCs upon oxidative stress [86] and has been linked to muscle loss with aging [87]. Glutamine is not considered to be an essential amino acid in homeostatic conditions; however, it can become essential in critical conditions such as cachexia [88] or aging, when muscle mass is more vulnerable [89].

Contrary to most of the publications on aging performed in serum and plasma [10, 13, 19, 22, 83, 84], a reduction of glutamate levels with age in RBCs (Figure 3) was found in our study. Previous studies have revealed an association between the decrease of glutamate levels and dementia, since it has an excitatory effect via N-methyl-d-aspartate receptor (NMDAR), that plays a critical role in synaptic plasticity and neuron survival. On the other hand, excessive NMDAR activity causes excitotoxicity and promotes cell death, underlying a potential mechanism of neurodegeneration in Alzheimer’s disease (AD). Therefore, it seems that glutamate has different effects in the brain depending on the receptors that are activated [90].

Proline and asparagine were the only non-essential amino acids whose levels increased with age (Figure 3), in agreement with results reported by Kouchiwa et al. [84] and Calvani et al. [83] in serum and plasma studies, respectively. High proline levels have been associated with the inhibition of the oxidative synthesis of this amino acid from pyrroline-5-carboxylate by NADPH and NADH consumption [9]. Thus, a proline increase with aging could have a protective effect in oxidative stress conditions. Asparagine is considered a non-essential amino acid, except when glutamine levels are low [91]. In this study, glutamine levels decreased with age, a finding that could reflect its consumption to generate asparagine.

RBC membrane, shape and turgor are also altered during aging, resulting in an increased osmotic fragility [92]. Interestingly, the decrease of the adipic acid levels observed in our study (Figure 3) could be associated with this phenomenon, as it has been described that dicarboxylic and tricarboxylic acids, such as adipic acid, might enter the RBC membrane, leaving the hydrophilic carboxylic groups outside, thus stabilizing the cell membrane and increasing osmotic resistance [93]. Additionally, we detected decreased betaine levels (Figure 3), a finding that has also been described to affect osmotic stability [94].

Interestingly, changes associated with metabolites transported by RBCs (amino acids) and the antioxidant system (glutathione) seem to take place earlier in life, as suggested by the comparison between the A1 and A2 subgroups, whereas alterations of the specific RBC metabolism (glycolysis, PPP, Luebering-Rapport shunt) only became apparent when comparing the A2 and A3 subgroups.

As for the impact of obesity on RBCs metabolism, a discrimination model between the BMI-1 and BMI-2 subgroups was also obtained (Figure 4A). To our knowledge, this is the first time this alteration is detected, as the only available studies on this issue had focused on specific alterations associated with the levels of fatty acids and folate acid [52, 53]. The S-plot (Figure 4B) revealed that the most significant changes associated with weight were an increase in the levels of some amino acids (glutamine, glycine an alanine), creatine and betaine, as well as a decrease in the concentrations of histidine, proline, 2,3-BPG and G1P. Supplementary Figure 5B displays a dot plot with the main metabolic pathways influenced by BMI (phenylalanine, glutathione, tryptophan, tyrosine, glutamine, glutamate, aspartate and alanine metabolism) after the integrative analysis of the metabolomics data.

The univariate analysis of the data (Figure 5), based on the evaluation of the changes in smaller subgroups (B1, B2, B3), revealed that BMI has the opposite effect of age in the concentrations of the amino acids. Thus, most of them showed an increase with BMI, whereas only proline, asparagine and histidine exhibited lower levels in obese individuals. Valine and leucine, BCAAs, increased with obesity, in agreement with the increase detected in blood plasma of these metabolites [48]. Glutamine, alanine and phenylalanine levels were also higher in obese individuals, as previously described in plasma/serum [7, 47, 48, 95], whereas glycine, whose levels also increased with BMI, showed the opposite trend in plasma studies [44, 47, 48]. These results concerning amino acids could perhaps be explained considering that obese subjects, suffering of overnutrition, have a relative IGF-1 deficiency that promotes catabolic pathways instead of protein synthesis [96]. Moreover, some studies suggest that the general increase of amino acids in obese individuals could be associated with the activation of the rapamycin complex1 (mTORC1) [97, 98]. Finally, glycine is involved in RBCs metabolism as a precursor of GSH [99], a compound that plays a central role in these cells, and it could explain the different levels of glycine found in RBCs and plasma samples. As opposed to the age-induced alterations, proline, asparagine and histidine levels declined with BMI. The levels of asparagine and histidine have also been reported to decrease with BMI in blood [43, 47, 49]; proline concentration, on the contrary, has been found to increase with BMI in plasma samples [44].

Furthermore, glucose levels increased with BMI in RBCs. Glucose blood levels are known to increase in obese subjects [43, 44]. Travis et al. described that elevated levels of glucose produce changes in RBCs metabolism [100]. Similarly, our results showed increased RBC creatine and phosphocreatine levels with BMI, as already reported in plasma [19, 44]. Taking into account that creatine can be obtained from food like meat or fish, this finding could be associated with a higher consumption of this kind of food by obese individuals [101].

Differences between genders were much less pronounced than alterations induced by aging or obesity. In fact, it was not possible to build any discriminant model between men and women. Previous studies have reported that gender has an effect on the levels of some blood metabolites, especially increased levels of amino acids have been detected in men [10, 22, 61, 84]. In our study, the univariate analysis of the metabolites detected in RBCs revealed that only threonine and ATP levels were significantly increased in men, while adipic acid seemed to be increased in women when compared with the levels found in men (Figure 6). Future studies including a higher number of samples could be required to detect subtle changes associated with gender.

The evaluation of correlations between age and BMI revealed diverse effects on the metabolic signature of RBCs. Good correlation models as a function of age were obtained, for both non-obese (BMI-1) and obese (BMI-2) individuals (Figure 7A). These analyses revealed that most of the metabolites contributing to the discrimination between age groups had the same trend in both BMI groups (Figure 7C). The pathway analysis (Supplementary Figure 5) showed that some of the alterations induced by age and BMI in RBCs matched in both studies. However, some of them exhibited opposite trends in BMI-1 and BMI-2 groups (e.g., several amino acids, 2,3-BPG, adipate, G1P, phosphoenolpyruvate, dimethylamine, formate), revealing different regulations of some pathways in obese and non-obese individuals. Regarding BMI correlations, a good prediction model of BMI was obtained for Age-1 subjects, whereas no model could be obtained for the Age-2 subgroup of individuals (Figure 7B). A possible explanation for this finding could be the reduced dispersion of BMI values in that subgroup (BMI range (Age-1) = 20.98/50.04; BMI range (Age-2) = 22.76/40.95). Overall, although several metabolites (e.g., asparagine, betaine, creatine, glutamate) showed opposite tendencies in each study, the results suggested that roughly half of the metabolites detected in RBCs exhibited similar trends with age on non-obese (BMI-1) individuals as BMI on younger individuals (Age-1).

Obesity appears to accelerate some age-associated changes, as individuals with higher BMI levels had lower levels of pyruvate and ATP and increased concentrations of aspartate, lactate and fumarate (Figure 7C). Interestingly, betaine levels increased with obesity, showing an opposite trend to that shown by age. Surprisingly, betaine supplementation has been shown to decrease body fat [102, 103], and low betaine levels in plasma has been previously associated with a higher cardiovascular risk [104]. Therefore, the increased betaine levels detected in RBCs does not seem to correlate with general higher betaine level in plasma or organs.

Interestingly, although HbA1c levels have been described to increase with age [105], a slight decrease of glucose levels was observed with age in RBCs. Finally, creatine and phosphocreatine levels showed an increase with BMI, opposite to the behavior observed with age. Asrin et al. found elevated levels of creatinine, degradation product of creatine, in obese female [106]. The increment of creatinine levels has been associated with raised blood pressure, leading to renal damage and cardiovascular disease [107]. Therefore, this result suggests that obese individuals, with high levels of creatine, could have an increased risk for this pathology.

Overall, this study represents the first systematic evaluation of the effect of age and obesity on the metabolomic profile of RBCs by NMR. The results revealed that both variables have a very relevant impact on RBC metabolism by up/down-regulating specific pathways associated with these cells. From the experimental point of view, the procedure is simple and robust, and only requires small volumes (<0.5ml) of blood. Significant changes were detected between different age-range groups reflecting a progressive alteration of the intrinsic metabolism of RBCs, as well as the metabolite pool transported by these cells, with age. Furthermore, obesity also induced significant alterations on the metabolic profile of RBCs; sometimes, in the same direction as those induced by aging. In other cases, alterations induced by obesity and age displayed opposite trends; so obesity could counteract the natural evolution of RBCs with age, and complicate the adaptation of the organism to senesce. These results would have to be validated in further studies using larger cohorts to confirm the findings as well as their clinical utility, as they could provide a new opportunity for assessing the impact of obesity and aging in RBCs.

## MATERIALS AND METHODS

### Solvents and reagents

Hank’s balanced salt solution was purchased from Gibco (Madrid, Spain), Ficoll Paque, methanol, chloroform and Na_2_HPO_4_ from Sigma-Aldrich (Madrid, Spain), D_2_O and deuterated trimethylsilylpropanoic (TSP) acid from Eurisotop (Gif sur Yvette, France). All the products were used as supplied. Cryogenics gases were provided by Air-Liquide (Valencia, Spain).

### Subjects

A cohort of 83 subjects, ages ranging between 19 and 71 years, were recruited at the Outpatient’s Department of the Endocrinology Service of Vall d’Hebron (Barcelona; 46 individuals) and the Dr. Peset University Hospitals (Valencia; 37 individuals) for this multicentric pilot study. Smokers, patients with type I or type II diabetes, cardiovascular complications or any other comorbidity, as well as individuals under pharmacological treatment were excluded from the study.

Depending on the analysis being performed, different age/BMI/gender subgroups were generated to ensure the groups were gender and/or age/BMI-matched. For that reason, not all the samples could be included in each individual comparative analysis. To evaluate the effect of age, two BMI-matched sub-groups were generated, “Age-1” (≤45 years) and “Age-2” (>45 years). The age cutoff was selected based on reports describing that individuals from 45-years old on start experiencing sarcopenia, a condition associated with insulin resistance, diabetes and metabolic comorbidity, morbidity and mortality [108]. Similarly, to characterize metabolic alterations associated with weight differences, samples were also classified in two age-matched groups corresponding to BMI<30 (“BMI-1”) and BMI≥30 (“BMI-2”), corresponding to non-obese and obese individuals, respectively [109]. Finally, women (“W”) and men (“M”) were separated in two different groups to assess the impact of gender. Both groups were age- and BMI-matched to avoid any influence of age and BMI on RBCs metabolism. Following multivariate statistical analysis, these groups were further divided in three age and BMI groups to better evaluate the precise evolution of the metabolite levels. The resulting groups were “A1” (19-40 years), “A2” (40-60 years) and “A3” (60-75 years) for the evaluation of the age effect, and “B1” (<30), “B2” (30-40) and “B3” (>40) for the characterization of the BMI effect on the metabolism of RBCs. The classification of the subgroups and the physiological and demographic characteristics of the individuals included in the study are summarized in Table 1 and Supplementary Table 5.

The study was conducted according to the guidelines established in the Declaration of Helsinki, and all procedures were approved by the Clinical Ethic Committees from the Vall d’Hebron (PR(AG)234/2015) and Dr. Peset University (CEIC140/14) Hospitals. Written informed consent was obtained from all the participants before sample collection.

### Isolation and storage of RBCs from peripheral blood

Peripheral blood was collected under fasting conditions, stored at 4° C and processed within the first hour. Biochemical analyses were performed at the biochemistry core facilities of the Vall d’Hebron and Dr. Peset University Hospitals to determine the plasma concentration of glucose, glycated haemoglobin (HbA1c), High Density Lipoprotein (HDL), Low Density Lipoprotein (LDL), triglycerides (TG), and insulin. BMI was calculated using the standard equations based on anthropometric information of the subjects [110].

5 mL of peripheral blood freshly extracted was poured carefully into a BD falcon tube containing 10 mL of Ficoll and left standing until the separation of a RBCs pellet in the bottom by gravity. Then, supernatant was discarded and the pellet was washed twice with 10 mL of cool PBS 1x solution in a centrifuge at 200 g and 4° C for 20 minutes without brakes. Cell purity was higher than 97% as determined by flow cytometry. For storage, a volume of ice-cold methanol equivalent to the amount of the cell pellet was added and the samples frozen directly at -80° C.

### Extraction of polar metabolites

Frozen samples were placed on ice and allowed to thaw for 5 min. Methanol was added until reaching a volume of 800 μL in total, and then, 800 μL of chloroform at 4° C were also added for the extraction. After 10 min, the samples were homogenized with a vortex, resuspended with a pipette and transferred to a plastic tube. For uniform cell breakage, the samples were placed in liquid nitrogen for 1 min and then allowed to thaw on ice for 2 min. This step was repeated twice. Afterwards, 1250 μL of distilled water and 1250 μL of chloroform were added and the sample was vortexed. Then, samples were centrifuged at 13000 g for 20 min at 4° C to facilitate the separation of the phases. The upper phase, containing polar metabolites in a mixture of water/methanol, was separated from the interphase and the lower chloroform phase, and then lyophilized for 2 hours. Extracts were stored at -80° C until the preparation of the samples for NMR analysis.

Samples for NMR analysis were placed on ice and allowed to thaw for 5 min. 550 μl of phosphate buffer (100 mM Na_2_HPO_4_ in D_2_O, pH 7.4), containing 0.1 mM deuterated TSP as internal standard, was added to the samples and the mixture was transferred to a 5 mm NMR tube. Samples were analyzed the same day and kept at 4° C.

### NMR experiments

NMR spectra were recorded at 27° C on a Bruker AVII-600 using a 5 mm TCI cryoprobe and processed using Topspin3.2 software (Bruker Biospin, Germany). ^1^H 1D noesy NMR spectra were acquired with 256 free induction decays (FIDs). 64k data points were digitalized over a spectral width of 30 ppm for an optimal baseline correction. A 4s relaxation delay was incorporated between FIDs and water presaturation was applied for aqueous samples. The FID values were multiplied by an exponential function with a 0.5 Hz line broadening factor. A water presaturation pulse of 25 Hz was applied throughout the relaxation delays to improve solvent suppression.

Total Correlation Spectroscopy (TOCSY) and multiplicity Heteronuclear Single Quantum Correlation (HSQC) were acquired for representative samples. For each TOCSY and HSQC experiment, 256 t1 increments and 32 and 96 FIDs were collected, respectively. The relaxation delay was set to 1.5s and the experiments were acquired in the phase-sensitive mode. TOCSY spectra were recorded using a standard MLEV-17 pulse sequence with mixing times (spin-lock) of 65 ms.

### Data analysis

1H-NMR signals from the spectra were assigned to their corresponding metabolites with the help of 2D NMR experiments, and information from spectral databases (Human Metabolome Database (HMBD) [6], Biological Magnetic Resonance Bank (BMRB) [111]). In ambiguous cases, the assignment was confirmed by spiking the spectra with reference compounds. Spectra were normalized to total intensity, excluding glucose and solvent signals, to minimize the differences in concentration and experimental error during the extraction process. In order to avoid signal overlapping, optimal integration regions were defined for each metabolite (Supplementary Table 1). Integration was performed using the GSD deconvolution function from MestReNovav12 (Mestrelab Research, Spain).

For multivariate statistical analyses, metabolite tables generated from spectral integration were univariate scaled, each value being divided by the standard deviation of each variable, and mean centered for an easier interpretation of the data and to take into account the variations of small signals. PCA, PLS and OPLS-DA were performed with SIMCA-P 14.0 (Umetrics, Sweden). The analysis of metabolites found to be relevant in the OPLS-DA model was carried out based on the evaluation of score plots, and S-plots, representing the modelled covariation, p [1], *versus* the modelled correlation, p(corr) [112]. PLS and OPLS-DA models were validated by permutation (n= 100) and ANalysis Of VAriance testing of Cross-Validated predictive residuals (CV-ANOVA) [113]. VIP values were calculated to capture the importance of the variables to the model, and those signals with values>1 were considered relevant in the analyses [114].

The statistical significance of the differences between the means of the groups was obtained using the Student t test in R (The R Foundation). A p-value <0.05 (confidence level 95%) was considered statistically significant.

## Supplementary Material

Supplementary Figures

Supplementary Tables
