# Supplementary material for: Metabolic footprint of aging and obesity in red blood cells
Source: Aging (Albany NY). 2021 Feb 19;13(4):4850–80. doi: 10.18632/aging.202693 (PMC7950240; doi:10.18632/aging.202693)
Supplement: Supplementary Figures [file aging-13-202693-s001.pdf]

SUPPLEMENTARY FIGURES

Supplementary Figures

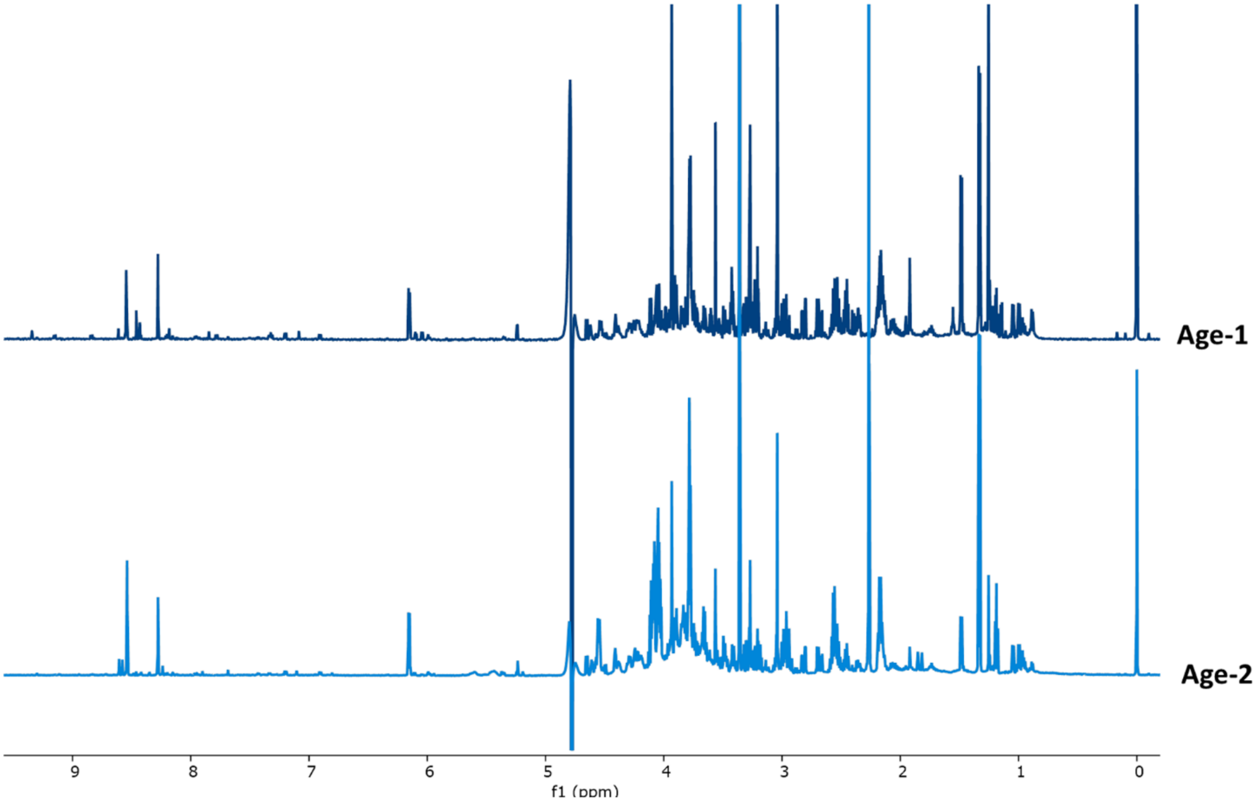

Supplementary Figure 1. Representative full 1H-NMR spectra corresponding to Age-1 and Age-2 groups.

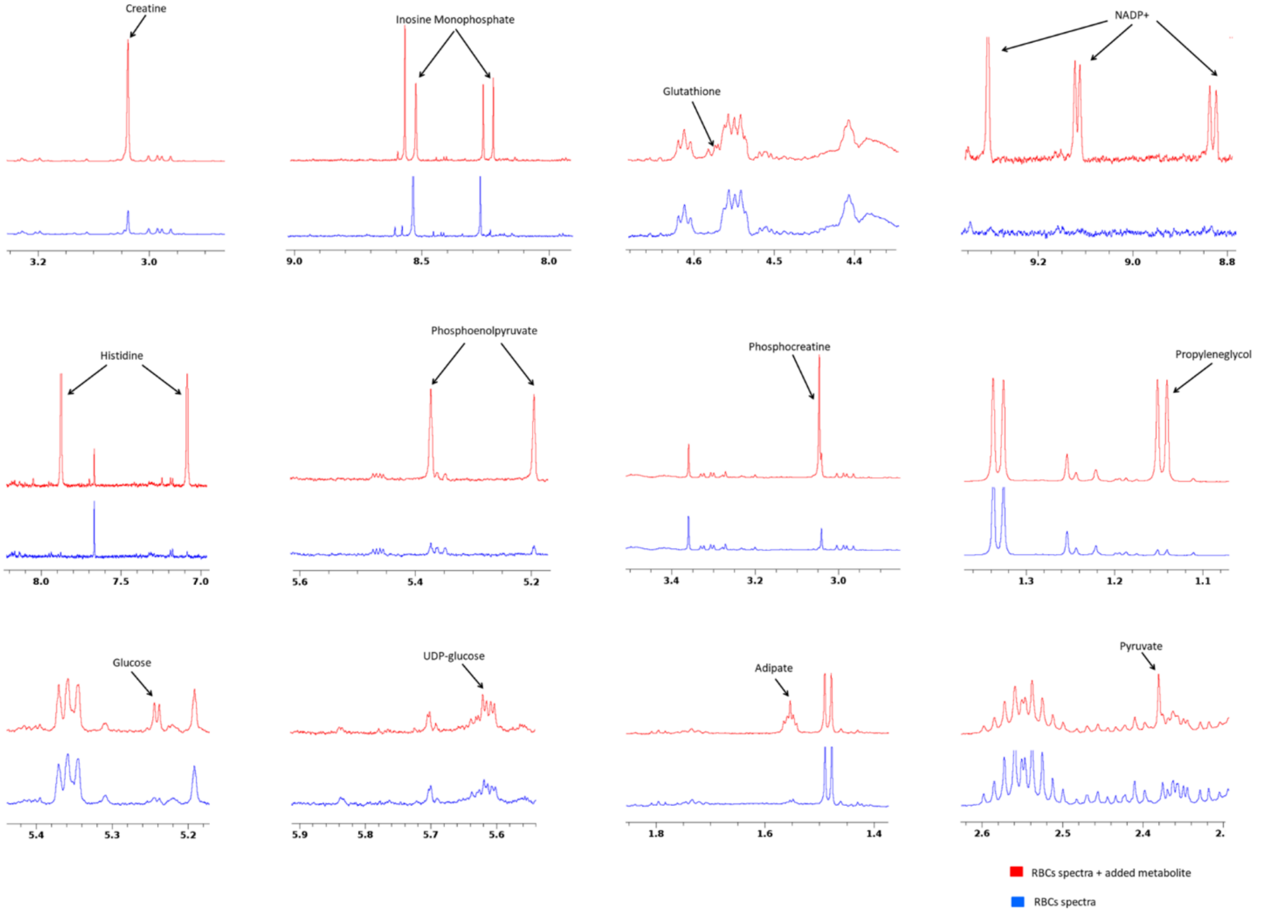

Supplementary Figure 2. Metabolite spiking NMR spectra with reference compounds. Red: ^{1}H-NMR spectrum of representative RBCs sample after the addition of 0.5 µL of the indicated metabolite (100mM). Blue: ^{1}H-NMR spectrum of the same sample before the addition of the standard metabolite. Spectra were acquired with a 600MHz spectrometer using 128 FIDs at 300K.

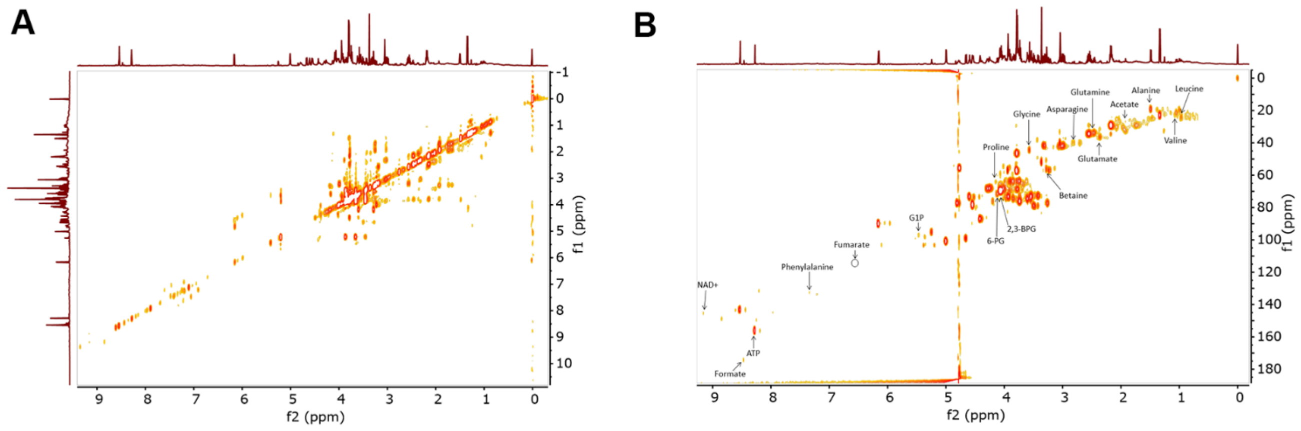

Supplementary Figure 3. (**A**) Total Correlation Spectroscopy (TOCSY) and (**B**) Heteronuclear Single Quantum Correlation (HSQC) NMR experiments acquired with a 600MHz spectrometer for representative samples. TOCSY and HSQC experiment were acquired using 256 t1 increments in both experiments, and 32 and 96 FIDs, respectively. The relaxation delay was set to 1.5s and the experiments were acquired in the phase-sensitive mode. TOCSY spectra were recorded using a standard MLEV-17 pulse sequence with mixing times (spin-lock) of 65 ms.

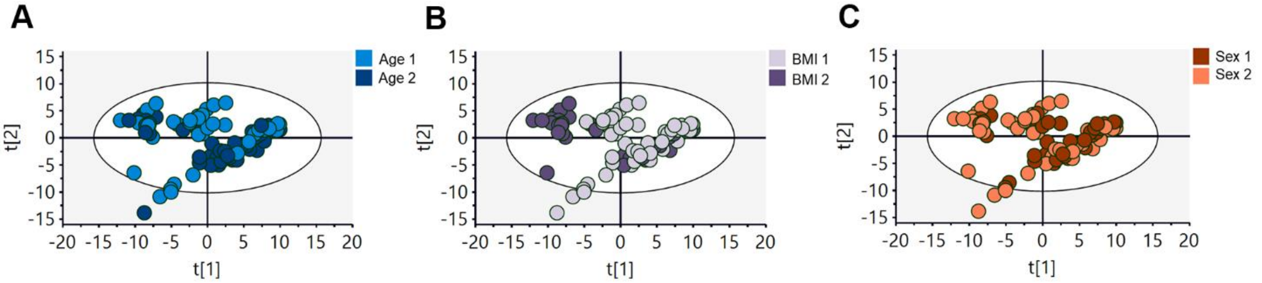

Supplementary Figure 4. PCA of all the samples. UV scaled, R2X= 0.872, Q2= 0.708. Score plot colored by age (**A**), BMI (**B**) and gender (**C**) groups.

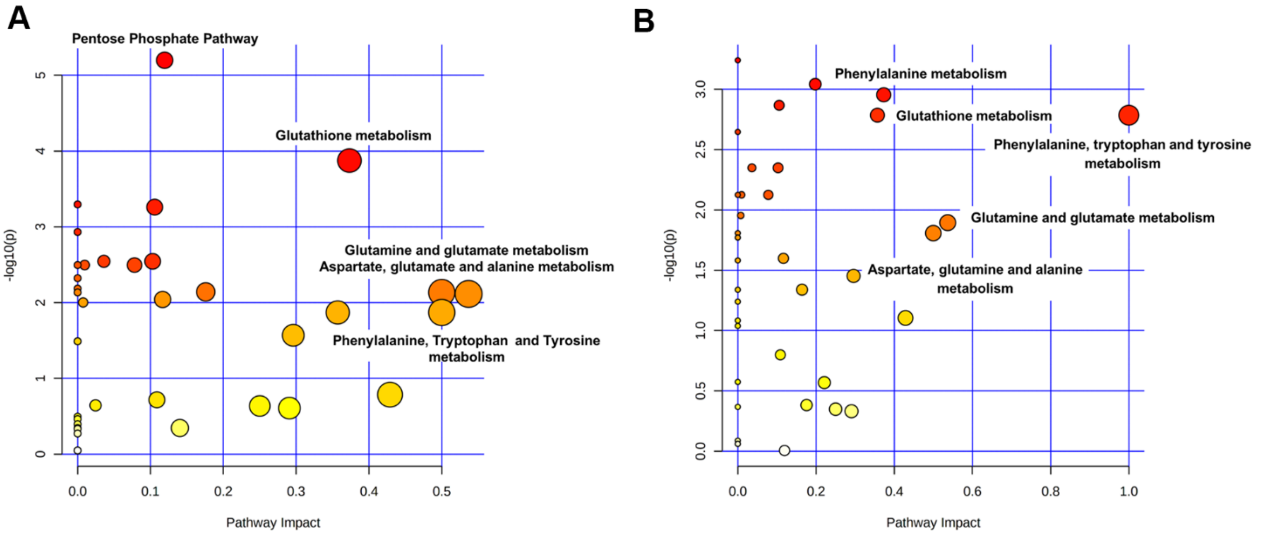

Supplementary Figure 5. Metabolic pathway analysis plot using Metaboanalyst. Plots display several metabolic pathway alterations induced by age (**A**, comparison between the Age-1 and Age-2 groups) and BMI (**B**, comparison between BMI-1 and BMI-2 groups). The x-axis represents the pathway impact value calculated from the pathway topological analysis, and the y-axis corresponds to the log of the p-value obtained from the pathway enrichment analysis. Each dot represents a unique metabolic pathway. The color depends on its p-value, red being the most significant, and the radius on the pathway impact values.
