# Supplementary material for: Metabolic footprint of aging and obesity in red blood cells
Source: Aging (Albany NY). 2021 Feb 19;13(4):4850–80. doi: 10.18632/aging.202693 (PMC7950240; doi:10.18632/aging.202693)
Supplement: Supplementary Tables [file aging-13-202693-s002.pdf]

SUPPLEMENTARY TABLES

Supplementary Table 1. Metabolites identified in RBCs and NMR integration regions used in this study.

Metabolite	Bucket (ppm)		Metabolite	Bucket (ppm)	
2,3-BPG*	4,0561	4.099	Histidine	7,073	7,12
2-HIV*	0,9119	0,9215	Histidine	7,834	7,865
3-HB*	1,1698	1,1726	Histidine	7,8881	7,911
3-HB	1,1791	1,1833	IMP*	8,23	8,24
3-MA*	0,86	0,911	IMP	8,573	8,588
6-PG*	3,83	3,838	Isoleucine	1,007	1,027
6-PG	4,099	4,104	Lactate	4,1041	4,123
6-PG	4,164	4,204	Leucine	0,94	0,946
Acetate	1,915	1,925	Leucine	0,951	0,9808
Acetoacetate	2,242	2,282	Lysine	1,5029	1,526
Adenosine	4,425	4,441	Lysine	1,526	1,532
Adenosine	4,445	4,466	Lysine	1,7	1,77
Adenosine	8,34	8,36	Lysine	1,871	1,909
Adipate	1,5327	1,5693	Lysine	1,9251	1,937
Adipate	2,195	2,206	Methionine	2,6451	2,65
Alanine	1,472	1,5	Myo-inositol	3,6221	3,632
AMP*	8,597	8,617	NAD+*	8,416	8,425
Asparagine	2,841	2,852	NAD+	8,839	8,854
Asparagine	2,852	2,865	NAD+	9,137	9,161
Asparagine	2,8651	2,879	NAD+	9,335	9,352
Aspartate	2,657	2,682	NADH	8,472	8,489
Aspartate	2,79	2,84	NADP+*	8,426	8,435
Aspartate	2,685	2,71	NADP+	8,81	8,823
ATP*	4,2281	4,25	NADP+	9,093	9,124
ATP	4,274	4,319	NADP+	9,292	9,308
ATP	4,595	4,632	Niacinamide	8,7	8,739
ATP	8,262	8,291	Niacinamide	8,936	8,952
Betaine	3,264	3,272	Nucleotides	5,937	5,957
Betaine	3,9020	3,908	Nucleotides	5,965	6,004
Choline	3,2031	3,214	Nucleotides	6,031	6,058
Creatine	3,035	3,045	Nucleotides	7,9271	7,947
Creatine	3,922	3,94	Ornithine	3,054	3,079
Dimethylamine	2,717	2,726	PEP*	5,032	5,045
Formate	8,454	8,469	PEP	5,126	5,138
Fumarate	6,514	6,527	PEP	5,364	5,377
Glucose	3,527	3,553	Phenylalanine	7,3351	7,35
Glucose	4,64	4,67	Phenylalanine	7,362	7,402
Glucose	5,232	5,248	Phenylalanine	7,3161	7,335
G1P*	5,445	5,475	Phosphocreatine	3,045	3,052
Glutamate	2,331	2,368	Proline	4,1231	4,128
Glutamine	2,435	2,483	Proline	4,133	4,155
Glycerol	3,669	3,673	Propylene glycol	1,1379	1,1565
Glycine	3,553	3,57	Pyroglutamate	2,388	2,397
GSH*	2,6	2,613	Pyruvate	2,368	2,3771
GSH	2,91	2,956	Succinate	2,4	2,41
GSH	2,9911	3	Threonine	4,251	4,272
GSH	4,569	4,591	Tyrosine	6,891	6,922
GSSG*	2,9561	2,968	UDP-glucose	5,5651	5,642
GSSG	2,9841	2,991	Valine	0,9865	1,0063
GSSG	3,001	3,011	Valine	1,035	1,0565
GSSG	3,29	3,333			
GSSG	3,769	3,778			

*2,3-BPG: 2,3-biphosphoglycerate; 2-HIV: 2-hydroxyisovalerate; 3-HB: 3-hydroxybutyrate; 3-MA: 3-methyladipate; 6-PG: 6-phosphogluconate; AMP: adenosine monophosphate; ATP: adenosine triphosphate; G1P: glucose 1-phosphate; GSH: reduced glutathione; GSSG: oxidized glutathione; IMP: inosine monophosphate; NAD+: nicotinamide adenine dinucleotide; NADP+: nicotinamide adenine dinucleotide phosphate; PEP: phosphoenolpyruvate.

Supplementary Table 2. Method of assignment of the significant metabolites of the study.

Metabolite	Assignment
2,3-BPG*	2D spectra
6-PG*	2D spectra
Acetate	2D spectra
Adipate	Spiking
Alanine	2D spectra
Asparagine	2D spectra
ATP*	2D spectra
Betaine	2D spectra
Creatine	Spiking
Formate	2D spectra
Fumarate	2D spectra
Glucose	Spiking
G1P*	2D spectra
Glutamate	2D spectra
Glutamine	2D spectra
GSH	Spiking
Glycine	2D spectra
Histidine	Spiking
IMP	Spiking
Leucine	2D spectra
NAD+*	2D spectra
NADP+*	Spiking
Phenylalanine	2D spectra
PEP*	Spiking
Phosphocreatine	Spiking
Proline	2D spectra
Propylene glycol	Spiking
Pyruvate	Spiking
UDP-Glucose	Spiking
Valine	2D spectra

*2,3-BPG: 2,3-biphosphoglycerate; 6-PG: 6-phosphogluconate; ATP: adenosine triphosphate; G1P: glucose 1-phosphate; GSH: reduced glutathione; IMP: inosine monophosphate; NAD+: nicotinamide adenine dinucleotide; NADP+: nicotinamide adenine dinucleotide phosphate; PEP: phosphoenolpyruvate.

Supplementary Table 3. Metabolite levels and univariate statistical analyses corresponding to different age comparisons.

			**Mean**			**SEM**			**Fold change (%)**			**P-value**		
**Metabolite**	**Bucket(ppm)**		**A1**	**A2**	**A3**	**A1**	**A2**	**A3**	**A1 *vs.* A2**	**A1 *vs.* A3**	**A2 *vs.* A3**	**A1 *vs.* A2**	**A1 *vs.* A3**	**A2 *vs.* A3**
2,3-BPG	4.056	4.099	26.771	25.115	31.150	2.346	1.603	1.821	6 ± 11	16 ± 11	24 ± 9	0.656	0.280	**0.020**
6-PG	4.099	4.104	0.909	1.776	2.682	0.134	0.343	0.410	95± 24	195 ± 21	51± 25	**0.007**	**4.24E-06**	0.102
Acetate	1.915	1.925	4.407	3.513	3.053	0.323	0.376	0.399	20 ± 13	31 ± 15	13 ± 17	0.109	**0.025**	0.412
Adipate	1.533	1.569	5.195	3.350	3.482	0.341	0.391	0.309	36± 13	33± 11	4± 15	**0.003**	**0.006**	0.799
Alanine	1.472	1.500	14.062	10.581	10.519	0.825	1.223	0.458	25± 13	25± 7	1± 12	**0.024**	**0.014**	0.965
Asparagine	2.841	2.852	0.747	0.759	0.960	0.052	0.121	0.081	2 ± 17	29 ± 11	26 ± 18	0.912	**0.038**	0.200
ATP	8.262	8.291	6.127	5.331	5.684	0.422	0.632	0.424	13 ± 14	7 ± 10	7 ± 14	0.300	0.551	0.659
Betaine	3.264	3.272	10.415	7.648	9.319	0.693	1.168	0.868	27 ± 17	11± 11	22± 18	**0.039**	0.386	0.278
Glucose	5.232	5.248	3.534	2.088	1.530	0.572	0.650	0.248	41 ± 35	57 ± 23	27 ± 35	0.109	**0.016**	0.462
G1P	5.445	5.475	0.977	1.444	1.562	0.119	0.217	0.120	48 ± 19	60± 14	8± 17	**0.048**	**0.008**	0.655
Glutamate	2.331	2.368	10.344	8.889	7.625	0.634	1.207	0.364	14 ± 15	26 ± 8	14 ± 14	0.248	**0.014**	0.362
Glutamine	2.435	2.483	12.292	9.274	10.400	0.591	1.186	0.533	25± 14	15± 7	12± 14	**0.015**	0.071	0.424
Glutathione	3.290	3.333	17.624	14.089	15.801	0.810	1.424	0.836	20 ± 11	10± 7	12± 11	**0.027**	0.207	0.334
Glycine	3.553	3.570	9.139	8.202	7.638	0.442	0.519	0.324	10 ± 8	16 ± 6	7 ± 8	0.217	0.052	0.387
IMP	8.230	8.240	0.250	0.266	0.496	0.019	0.041	0.089	6 ± 17	98 ± 19	86 ± 24	0.690	**3.06E-04**	**0.021**
Lactate	4.104	4.123	6.603	7.344	7.678	0.425	0.36	0.549	11 ± 8	16 ± 10	5 ± 9	0.284	0.171	0.605
NAD+	9.335	9.352	0.685	0.507	0.455	0.056	0.061	0.033	26 ± 15	34 ± 11	10 ± 14	0.063	**0.019**	0.488
PEP	5.364	5.377	0.397	0.343	0.474	0.045	0.043	0.033	14 ± 17	19 ± 13	38 ± 14	0.469	0.326	**0.029**
Proline	4.133	4.155	2.580	3.149	3.047	0.118	0.239	0.142	22 ± 9	18± 7	3± 9	**0.021**	**0.033**	0.730
Pyruvate	2.368	2.377	2.154	2.218	1.881	0.073	0.112	0.065	3 ± 6	13 ± 5	15 ± 6	0.642	**0.029**	**0.015**
UDP-glucose	5.565	5.642	1.321	1.890	2.779	0.100	0.342	0.209	43 ± 20	110± 11	47± 20	**0.039**	**2.98E-08**	**0.036**
Valine	1.035	1.057	5.163	4.039	4.265	0.299	0.434	0.246	22 ± 12	17± 8	6± 12	**0.041**	0.087	0.671

*2,3-BPG: 2,3-biphosphoglycerate; 6-PG: 6-phosphogluconate; ATP: adenosine triphosphate; G1P: glucose 1-phosphate; IMP: inosine monophosphate; NAD+: nicotinamide adenine dinucleotide; PEP: phosphoenolpyruvate.

Supplementary Table 4. Metabolite levels and univariate statistical analyses corresponding to the comparison of different BMI subgroups.

			Mean			SEM			Fold change (%)			P-value		
**Metabolite**	**Bucket (ppm)**		**B1**	**B2**	**B3**	**B1**	**B2**	**B3**	**B1 *vs* B2**	**B1 *vs.* B3**	**B2 *vs* B3**	**B1 *vs* B2**	**B1 *vs* B3**	**B2 *vs* B3**
2,3-BPG*	4.056	4.099	25.214	20.140	18.214	1.582	2.156	2.365	20 ± 12	28 ± 14	10 ± 17	0.067	**0.030**	0.576
6-PG*	4.099	4.104	2.868	3.623	3.529	0.221	0.217	0.128	26± 10	23± 9	3± 7	**0.022**	**0.028**	0.717
Adipate	1.533	1.569	3.606	3.328	2.613	0.384	0.418	0.528	8 ± 16	28 ± 23	21 ± 24	0.627	0.138	0.300
Alanine	1.472	1.5	11.011	17.781	18.116	1.046	1.482	1.563	61± 13	65± 13	2± 12	**0.001**	**0.001**	0.878
Asparagine	2.841	2.852	0.800	0.580	0.581	0.110	0.122	0.136	28± 25	27± 27	0± 31	0.191	0.220	1.000
Betaine	3.264	3.272	8.370	12.423	13.782	1.014	1.276	1.315	48 ± 16	65 ± 15	11 ± 14	**0.018**	**0.003**	0.464
Creatine	3.035	3.045	14.705	20.910	24.400	1.992	1.886	2.447	42 ± 16	66 ± 17	17 ± 13	**0.031**	**0.004**	0.267
Formate	8.454	8.469	0.632	1.034	0.992	0.078	0.132	0.130	64± 18	57± 18	4± 18	**0.013**	**0.023**	0.823
Fumarate	6.514	6.527	0.205	0.306	0.316	0.029	0.035	0.034	49 ± 18	54 ± 18	3 ± 16	0.060	**0.038**	0.828
Glucose	5.232	5.248	2.182	3.919	5.728	0.563	0.662	1.262	80± 31	163± 34	46± 28	0.054	**0.015**	0.213
Glutamine	2.435	2.483	9.381	12.874	13.592	0.928	0.514	1.121	37 ± 11	45 ± 13	6 ± 9	**0.002**	**0.007**	0.564
Glycine	3.553	3.570	8.306	11.280	12.174	0.478	1.079	1.057	36± 11	47± 10	8± 13	**0.017**	**0.002**	0.558
Leucine	0.951	0.981	6.061	7.615	7.354	0.612	0.431	0.564	26± 12	21± 13	3± 10	**0.046**	0.130	0.715
NAD+*	9.335	9.352	0.493	0.659	0.626	0.058	0.052	0.054	34 ± 14	27 ± 15	5 ± 12	**0.040**	0.102	0.654
NADP+*	9.292	9.308	0.407	0.506	0.509	0.041	0.031	0.050	24 ± 12	25 ± 14	1 ± 12	0.160	0.171	0.958
Phenylalanine	7.335	7.350	0.573	0.678	0.664	0.054	0.040	0.044	18 ± 11	16 ± 12	2 ± 9	0.127	0.200	0.815
Phosphocreatine	3.045	3.052	2.809	3.425	3.562	0.272	0.179	0.262	22 ± 11	27 ± 12	4 ± 9	0.068	0.054	0.668
Proline	4.133	4.155	3.032	2.842	2.485	0.193	0.093	0.179	6± 7	18± 10	13± 8	0.382	**0.047**	0.082
Propyleneglycol	1.138	1.157	1.923	1.166	0.881	0.307	0.187	0.195	39± 23	54± 27	24± 27	**0.043**	**0.007**	0.300
Pyruvate	2.368	2.377	2.127	1.995	1.938	0.139	0.084	0.107	6± 8	9± 9	3± 7	0.403	0.282	0.675
Valine	1.035	1.057	4.324	5.618	5.498	0.389	0.312	0.468	30± 11	27± 12	2± 10	**0.014**	0.063	0.832

*2,3-BPG: 2,3-biphosphoglycerate; 6-PG: 6-phosphogluconate; NAD+: nicotinamide adenine dinucleotide; NADP+: nicotinamide adenine dinucleotide phosphate.

Supplementary Table 5. Classification of the individuals included in the study.

Sample	Hospital	Gender	Age	BMI	Age-1/Age-2	A1/A2/A3	BMI-1/BMI-2	B1/B2/B3
ERI 79	Peset	W*	26	20.60	-	A1	BMI-1	B1
ERI 84	Peset	W	21	23.10	Age-1	A1	-	-
ERI 85	Peset	W	19	21.10	Age-1	A1	-	-
ERI 87	Peset	**W**	25	24.20	Age-1	A1	-	-
ERI 91	Peset	**W**	24	24.40	Age-1	A1	-	-
ERI 93	Peset	**W**	45	22.70	Age-1	A2	BMI-1	B1
ERI 94	Peset	**W**	50	35.80	Age-2	-	BMI-2	B2
ERI 96	Peset	**W**	39	21.70	-	A1	BMI-1	-
ERI 100	Peset	W	30	19.60	-	A1	BMI-1	B1
ERI 104	Peset	W	19	24.00	Age-1	A1	-	-
ERI 107	Peset	W	42	-	Age-1	A2	-	-
ERI 112	Peset	**W**	27	27.00	Age-1	A1	BMI-1	-
ERI 117	Peset	**W**	39	-	Age-1	-	BMI-2	-
ERI 118	Peset	**W**	48	40.73	-	-	BMI-2	B3
ERI 121	Peset	W	40	39.00	Age-1	-	BMI-2	B2
ERI 122	Peset	**W**	40	31.54	Age-1	-	BMI-2	B2
ERI 123	Peset	W	40	32.60	Age-1	-	BMI-2	B2
ERI 124	Peset	**W**	63	32.80	Age-2	-	BMI-2	B2
ERI 128	Peset	W	32	33.35	Age-1	-	BMI-2	B2
ERI 129	Peset	W	45	45.78	Age-1	-	BMI-2	B3
ERI 130	Peset	W	38	42.80	Age-1	-	BMI-2	B3
ERI 132	Peset	W	37	50.04	Age-1	-	BMI-2	B3
ERI 135	Peset	W	58	49.09	-	-	BMI-2	B3
ERI 137	Peset	W	49	42.08	-	-	BMI-2	B3
ERI 138	Peset	W	35	37.70	Age-1	-	BMI-2	B2
ERI 139	Peset	**W**	52	48.80	-	-	BMI-2	B3
ERI 162	Vall d'Hebron	**W**	65	23.63	Age-2	A3	BMI-1	B1
ERI 163	Vall d'Hebron	**W**	58	33.77	Age-2	-	BMI-2	B2
ERI 168	Vall d'Hebron	**W**	70	31.96	Age-2	-	BMI-2	B2
ERI 169	Vall d'Hebron	**W**	69	29.91	Age-2	A3	BMI-1	B1
ERI 171	Vall d'Hebron	**W**	58	41.02	-	-	BMI-2	B3
ERI 172	Vall d'Hebron	**W**	63	47.30	-	-	BMI-2	B3
ERI 173	Vall d'Hebron	**W**	55	43.97	-	-	BMI-2	B3
ERI 174	Vall d'Hebron	**W**	58	48.60	Age-2	-	BMI-2	B3
ERI 177	Vall d'Hebron	W	58	50.22	-	-	BMI-2	B3
ERI 180	Vall d'Hebron	**W**	58	45.77	-	-	BMI-2	B3
ERI 314	Vall d'Hebron	**W**	63	-	Age-2	A3	BMI-1	-
UOM 1	Vall d'Hebron	**W**	54	22.76	Age-2	A2	BMI-1	B1
UOM 2 ERI	Vall d'Hebron	W	39	18.96	-	A1	BMI-1	B1
UOM 3	Vall d'Hebron	**W**	45	24.16	Age-1	A2	BMI-1	B1
UOM 4	Vall d'Hebron	W	34	20.98	Age-1	A1	BMI-1	-
UOM 13	Vall d'Hebron	**W**	59	28.20	Age-2	A2	BMI-1	-
UOM 14	Vall d'Hebron	**W**	31	19.14	-	A1	BMI-1	-
UOM 16	Vall d'Hebron	**W**	68	25.51	Age-2	A3	BMI-1	-
UOM 21	Vall d'Hebron	**W**	37	23.87	Age-1	A1	BMI-1	-
UOM 26	Vall d'Hebron	**W**	31	19.16	-	A1	BMI-1	-
ERI 81	Peset	**M***	52	23.70	-	A2	BMI-1	B1
ERI 83	Peset	**M**	25	23.40	Age-1	A1	-	-
ERI 90	Peset	**M**	25	23.90	Age-1	A1	-	-
ERI 97	Peset	**M**	47	23.40	Age-2	A2	BMI-1	B1
ERI 109	Peset	**M**	25	28.80	Age-1	A1	-	-
ERI 111	Peset	**M**	39	-	Age-1	A1	BMI-1	-
ERI 120	Peset	**M**	53	38.60	Age-2	-	BMI-2	B2
ERI 125	Peset	**M**	53	45.06	-	-	BMI-2	B3
ERI 131	Peset	**M**	54	43.30	-	-	BMI-2	B3
ERI 133	Peset	**M**	45	30.93	Age-1	-	BMI-2	B2
ERI 134	Peset	**M**	46	35.01	Age-2	-	BMI-2	B2

ERI 160	Vall d'Hebron	**M**	70	28.08	Age-2	A3	BMI-1	B1
ERI 161	Vall d'Hebron	**M**	61	31.40	Age-2	A3	BMI-2	B2
ERI 164	Vall d'Hebron	**M**	64	25.51	Age-2	A3	BMI-1	B1
ERI 165	Vall d'Hebron	**M**	52	29.41	Age-2	A2	BMI-1	B1
ERI 166	Vall d'Hebron	**M**	63	30.11	Age-2	-	BMI-2	B2
ERI 167	Vall d'Hebron	**M**	57	28.40	Age-2	A2	BMI-1	B1
ERI 170	Vall d'Hebron	**M**	71	30.03	Age-2	-	BMI-2	B2
ERI 175	Vall d'Hebron	M	61	26.98	Age-2	A3	BMI-1	B1
ERI 176<br>UOM 3	Vall d'Hebron	**M**	60	30.00	Age-2	-	BMI-2	B2
ERI 178	Vall d'Hebron	**M**	52	36.57	Age-2	-	BMI-2	B2
ERI 179	Vall d'Hebron	**M**	55	40.95	Age-2	-	BMI-2	B3
ERI 312	Vall d'Hebron	**M**	59	-	Age-2	A2	BMI-1	-
ERI 313	Vall d'Hebron	**M**	65	-	Age-2	A3	BMI-1	-
ERI 315	Vall d'Hebron	M	54	-	Age-2	A2	BMI-1	-
ERI 316	Vall d'Hebron	M	60	-	Age-2	A3	BMI-1	-
UOM 5	Vall d'Hebron	M	55	29.04	Age-2	A2	BMI-1	B1
UOM 10	Vall d'Hebron	**M**	38	22.38	Age-1	A1	BMI-1	-
UOM 12	Vall d'Hebron	**M**	42	24.22	Age-1	A2	BMI-1	B1
UOM 15	Vall d'Hebron	**M**	70	22.85	Age-2	A3	BMI-1	-
UOM 17	Vall d'Hebron	M	33	27.75	Age-1	A1	BMI-1	-
UOM 18	Vall d'Hebron	**M**	29	19.81	-	A1	BMI-1	-
UOM 19	Vall d'Hebron	M	28	29.04	Age-1	A1	BMI-1	-
UOM 20	Vall d'Hebron	M	26	20.38	-	A1	BMI-1	-
UOM 22	Vall d'Hebron	M	24	32.69	Age-1	A1	BMI-2	-
UOM 23	Vall d'Hebron	M	26	22.04	Age-1	A1	BMI-1	-
UOM 24	Vall d'Hebron	**M**	31	20.67	-	A1	BMI-1	-

*M = Men, W = Women; M and W in bold are the selected samples for gender analysis.
